# Analysis of routine blood parameters in patients with amyotrophic lateral sclerosis and evaluation of a possible correlation with disease progression—a multicenter study

**DOI:** 10.3389/fneur.2022.940375

**Published:** 2022-07-27

**Authors:** Nora Hertel, Magdalena Kuzma-Kozakiewicz, Marta Gromicho, Julian Grosskreutz, Mamede de Carvalho, Hilmi Uysal, Reinhard Dengler, Susanne Petri, Sonja Körner

**Affiliations:** ^1^Department of Neurology, Hannover Medical School, Hanover, Germany; ^2^Department of Neurology, Medical University of Warsaw, Warsaw, Poland; ^3^Institute of Physiology-Instituto de Medicina Molecular, Faculdade de Medicina, Universidade de Lisboa, Lisbon, Portugal; ^4^Department of Neurology, University Hospital Lübeck, Lübeck, Germany; ^5^Department of Neurology, Faculty of Medicine, Akdeniz University, Antalya, Turkey; ^6^Center for Systems Neuroscience (ZSN), Hanover, Germany

**Keywords:** amyotrophic lateral sclerosis, creatine kinase, albumin, creatinine, lipids, disease progression, biomarker

## Abstract

**Objective:**

Amyotrophic lateral sclerosis (ALS) pathogenesis is still unclear, its course is considerably variable, and prognosis is hard to determine. Despite much research, there is still a lack of easily accessible markers predicting prognosis. We investigated routine blood parameters in ALS patients regarding correlations with disease severity, progression rate, and survival. Additionally, we analyzed disease and patients' characteristics relating to baseline blood parameter levels.

**Methods:**

We analyzed creatine kinase (CK), albumin (ALB), creatinine (CREA), total cholesterol (TC), high-density lipoprotein cholesterol (HDL), low-density lipoprotein cholesterol (LDL), and triglycerides (TG) levels around time of diagnosis in 1,084 ALS patients. We carried out linear regression analyses including disease and patients' characteristics with each blood parameter to detect correlations with them. Linear regression models were performed for ALSFRS-R at study entry, its retrospectively defined rate of decay and prospectively collected progression rate. Different survival analysis methods were used to examine associations between blood parameters and survival.

**Results:**

We found higher CK (*p*-value 0.001), ALB (*p*-value <0.001), CREA (*p*-value <0.001), and HDL levels (*p*-value 0.044) at time of diagnosis being associated with better functional status according to ALSFRS-R scores at study entry. Additionally, higher CREA levels were associated with lower risk of death (*p*-value 0.003).

**Conclusions:**

Our results indicate potential of CK, ALB, CREA, and HDL as disease severity or progression markers, and may also provide clues to ALS pathogenesis. However, these values are highly dependent on other variables, and further careful, longitudinal analyses will be necessary to prove the relevance of our findings.

## Introduction

Amyotrophic lateral sclerosis is a motor neuron disease characterized by degeneration of upper and lower motor neurons. Depending on the region of onset, initial symptoms are fasciculations, weakness of limb muscles, functional impairment, dysarthria, and dysphagia. The majority of patients present with limb onset, one third with bulbar onset and up to 5% with respiratory symptoms (1). As the disease progresses, patients usually die within 3–5 years, mostly due to respiratory failure (2). With an incidence of 2.16 per 100.000 person-years in Europe, ALS is the most common adult-onset motor neuron disease (3). Only 5–10% of the cases are caused by monogenic mutations with autosomal-dominant inheritance, while the remaining 90–95% of the patients are diagnosed with sporadic ALS (1). More than 20 ALS-associated genes have been discovered so far (2), but the pathogenesis of sporadic ALS has still not been completely understood. Despite intensive research, easily accessible markers providing information about disease severity or progression rate are lacking. This is of particular interest as there is an enormous heterogeneity regarding different ALS phenotypes and the rate of progression.

Increased creatine kinase (CK) levels have been found in 38–52% of ALS patients (4–16). There is evidence for a role of impaired energy metabolism in motor neuron degeneration (17, 18). Since CK is crucial for maintaining creatine phosphate, an important energy carrier in muscle cells, its potential as biomarker for ALS seems plausible (19). Previous studies have so far provided rather controversial results as some researchers found associations with disease severity (13, 20), while others did not (7–9). Its potential as biomarker predicting survival also remains unclear, since study results vary here as well (4, 6, 7, 9, 10, 12, 15, 16, 21–23).

Fewer studies have investigated albumin (ALB) levels in ALS, even though albumin is an acute phase protein, and neuroinflammation has been identified as an ALS pathomechanism (24). It can reflect the nutritional status of ALS patients, which worsens in the disease course and represents a negative prognostic factor (25). Previous studies showed a correlation between lower ALB levels and shorter survival (26–28), and there is substantial evidence that ALB correlates with disease severity and functional decline (28).

Since muscular weakness is one of the main symptoms in ALS, biomarkers of muscle metabolism hold potential for predicting prognosis or progression (1). Creatinine (CREA) is a breakdown product of creatine, which is involved in the energy metabolism of muscles and is constantly released from muscle cells (19). Since it correlates with lean muscle mass, it might be a promising biomarker candidate (29). Most studies found lower CREA levels in ALS patients related to shorter survival (22, 23, 26, 27, 30, 31) and faster disease progression (22, 27, 31–35), yet again results are inconsistent (13, 21, 36).

Higher resting energy expenditure and a decline of body mass index (BMI) were associated with worse outcome (37–39), which indicates a potential of metabolic parameters, such as lipids, as prognostic biomarkers of ALS. Even though lipid levels are probably among the most extensively studied blood biomarkers, the literature could not be any more inconsistent. Repeatedly, researchers found associations between lipid markers and severity, progression, or survival, but there is no common agreement which parameter is most suitable, neither about the direction of correlation. A consensus regarding a link between history of dyslipidemia and higher or lower risk of ALS and patients' survival has not yet been established.

Up to date, neurofilament levels in cerebrospinal fluid and blood seem to be the most promising ALS biomarkers, especially regarding disease progression (40). Analysis of blood biomarkers is easier and less invasive and therefore a more suitable option for assessing disease progression or prognosis and neurofilaments cannot yet be determined comprehensively in every laboratory. We therefore aimed to investigate if routine blood parameters such as CK, ALB, CREA, total cholesterol (TC), high-density lipoprotein cholesterol (HDL), low-density lipoprotein cholesterol (LDL), and triglyceride (TG) correlate with patient disease characteristics or can reflect the severity and predict prognosis. Reliable blood biomarkers may also generate further information about ALS pathogenesis and be useful for patient monitoring in clinical trials.

## Materials and methods

We conducted a multicenter study with five study centers in Germany, Portugal, Poland, and Turkey. Data of 1,084 ALS-patients who presented themselves at one of the hospitals participating in the OnWebDUALS-consortium between July 2015 and May 2020 (Hannover Medical School, Faculty of Medicine Lisbon, Medical University Warsaw, Friedrich Schiller University Jena, Akdeniz University Faculty of Medicine, Antalya) were collected. Patients either visited the outpatient clinics or were admitted to the inpatient area for diagnostic examinations or complaints regarding their disease. All patients with at least one blood value of interest available at the time of diagnosis were included in the study. 83.8% met the El Escorial criteria for definite, probable, possible, or probable laboratory-supported ALS. Except 15 patients with primary lateral sclerosis, all patients could be diagnosed with ALS according to the recently proposed Gold Coast criteria (41, 42).

After patients had signed an informed consent form, they were included into the OnWebDUALS registry and interviewed via a standardized questionnaire developed by the OnWebDUALS-consortium (43). This questionnaire included information about individual disease characteristics and history (clinical symptoms, region of onset, disease severity), results of EMG and blood tests, medication, medical and family history as well as lifestyle features. To further investigate the impairment of the patients, we used the revised ALS functional rating scale (ALSFRS-R), which is widely established for monitoring functional status (44).

**Figure 1:**
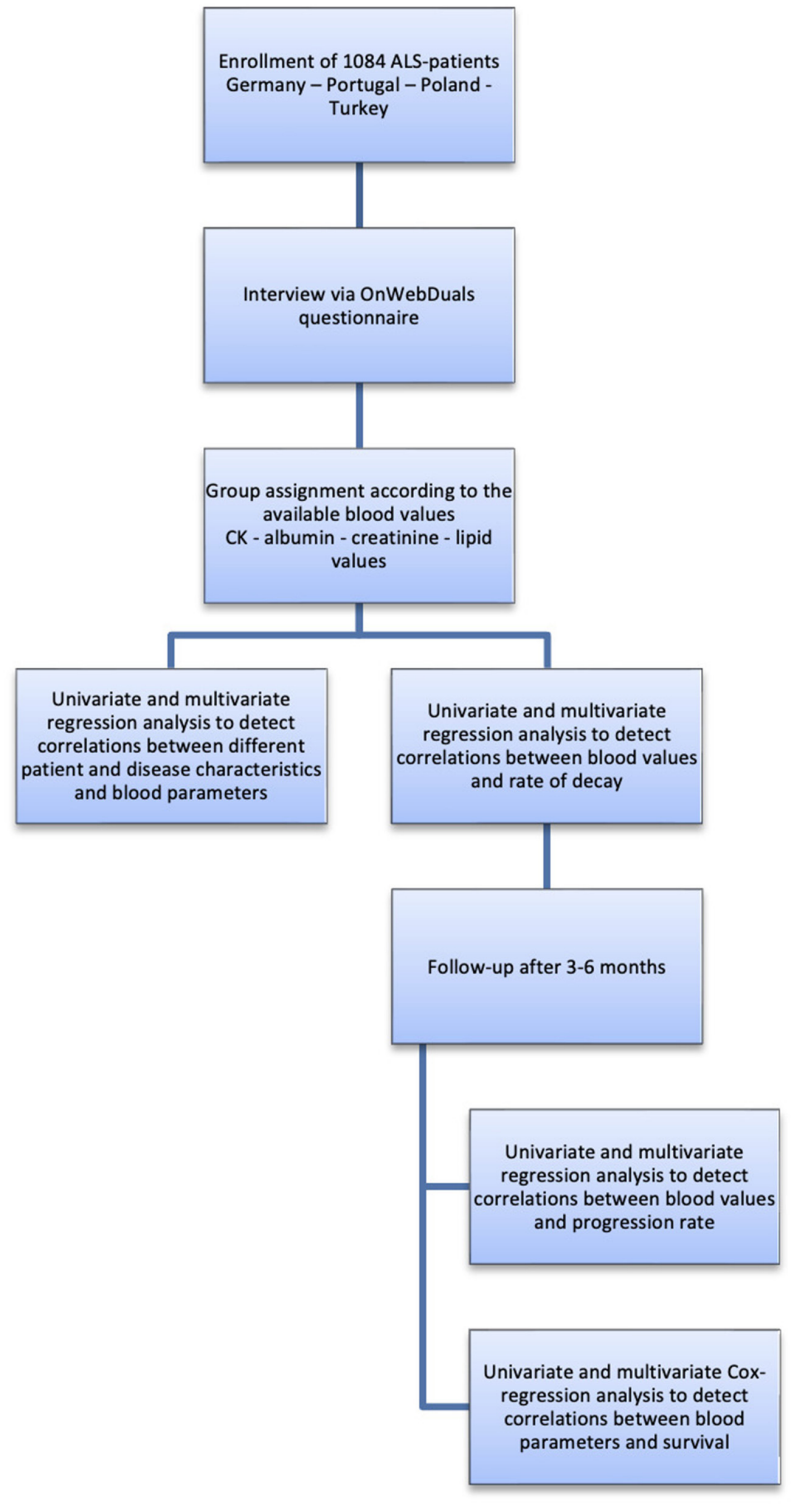
Research flow chart.

Blood samples were taken either at time point of inclusion or blood values from previous visits were used. Results of blood tests were also obtained from the patients' family physicians. We only included values taken within up to 1 year before or after the diagnosis. In addition to these retrospectively acquired data, the centers Hanover and Lisbon also collected a second ALSFRS-R score 3–6 months after inclusion which was used for prospective calculation of the progression rate. Follow-up was either carried out in person, per phone, per letter or per e-mail (Figure 1).

For the analyses regarding the association of certain blood values and disease characteristics, patients were grouped according to which blood parameters were available in each patient. The characteristics of the groups are presented in Tables 1.1, 1.2.

**Table 1.1:** Description of patient's characteristics according to blood parameter-group.

	**Complete cohort**		**CK (U/l)**		**Albumin (mg/dl)**		**Creatinine (mg/dl)**	
	***n***		***n***		***n***		***n***	
Female (*n*)	1,084	41.6% (451)	817	41.7% (341)	522	42.5% (222)	991	41.9% (415)
Age at diagnosis (SD)	1,058	61.07 (12.8)	792	60.55 (13.24)	503	61.27 (13.01)	965	61.23 (12.9)
Region of onset (*n*)	1,083		816		522		990	
Limb		70.5% (764)		72.1% (589)		72.2% (377)		70.0% (694)
Bulbar		25.7% (279)		25.0% (204)		24.1% (126)		25.9% (257)
Thoracic		2.6% (28)		2.2% (18)		2.7% (14)		2.8% (28)
Dyscogn		1.1% (12)		0.6% (5)		1.0% (5)		1.1% (11)
Diagnostic delay (SD)	1,057	17.51 (23.05)	791	17.53 (23.65)	503	18.24 (22.43)	964	17.83 (23.69)
Predominant UMN (*n*)	1,035	27.6% (299)	781	26.3% (215)	501	31.0% (162)	942	28.1% (278)
Predominant LMN (*n*)	1,035	74.5% (808)	781	76.1% (622)	501	74.5% (389)	942	73.9% (732)
BP (SD)			817	243.42 (234.29)	522	4.11 (0.52)	991	0.76 (0.29)

*BP, blood parameter; SD, standard deviation*.

**Table 1.2:** Description of patient's characteristics according to blood parameter-group.

	Total cholesterol (mg/dl)		HDL (mg/dl)		LDL (mg/dl)		Triglyceride (mg/dl)	
	***n***		***n***		***n***		***n***	
Female (*n*)	704	43.0% (303)	621	41.9% (260)	616	42.2% (260)	669	42.4% (282)
Age at diagnosis (SD)	688	60.31 (12.93)	602	60.17 (12.95)	599	60.25 (12.94)	650	60.42 (12.81)
Region of onset (*n*)	704		621		616		669	
Limb		71.3% (502)		72.0% (447)		71.8% (442)		70.7% (473)
Bulbar		25.9% (182)		25.0% (155)		25.2% (155)		26.2% (175)
Thoracic		2.0% (14)		2.3% (14)		2.3% (14)		2.2% (15)
Dyscogn		0.9% (6)		0.8% (5)		0.8% (5)		0.9% (6)
Diagnostic delay (SD)	688	17.93 (23.56)	602	18.62 (24.23)	599	18.6 (24.31)	650	18.25 (23.64)
Predominant UMN (*n*)	676	27.3% (192)	591	26.7% (166)	587	26.6% (164)	638	27.1% (181)
Predominant LMN (*n*)	676	77.4% (545)	591	77.5% (481)	587	77.8% (479)	638	76.5% (512)
BP (SD)	704	206.27 (49.28)	621	54.91 (18.23)	616	128.25 (42.4)	616	130.44 (68.39)

*BP, blood parameter; SD, standard deviation*.

The study has been approved by the ethical committees of all participating centers.

### Statistical analysis

Continuous variables were checked for normal distribution by using boxplots and described with their mean and standard deviation. For categorial variables the percentage and absolute number of patients was displayed. Region of onset [containing limb, bulbar, thoracic, and dyscognition (=dementia symptoms or behavioral anomalies) onset] was transformed into dummy variables for the inclusion in regression models, where limb onset served as reference category. The rate of decay was defined as the difference between the maximal possible ALSFRS-R score of 48 (=completely healthy) and the score of the patient at study entry divided by the time since onset in months [(48—ALSFRS-R at study entry)/time since onset]. The calculation of the rate of decay has been established in several studies before (45, 46). The rate of decay retrospectively gives a hint about the speed of disease progression until study entry. For patients with follow-up data regarding ALSFRS-R scores (Hanover and Lisbon cohorts) also the “progression rate” was calculated. The progression rate was defined as the difference between the ALSFRS-R score at study entry and at follow-up divided by the time between those evaluations in months [(ALSFRS-R1 – ALSFRS-R2)/time between study entry and follow-up] and thus represents a prospective progression parameter.

To detect correlations between different patient and disease characteristics and the blood parameters, we separately performed linear regression models for each blood parameter as dependent variable. First, we calculated univariate regression models for the variables gender, age at diagnosis, region of onset, UMN or LMN predominance at onset, upper and lower limb involvement at onset, rate of decay, diagnostic delay, smoking status, physical exercise (PE) as well as rural or urban area of living in the last 5 years and before. Since statins have an influence on blood lipids and the CK level, we included statin intake additionally in the analyses for these blood parameters. Variables with a *p*-value <0.2 in the univariate analysis were included in a multivariate model. Subsequently, we used backward selection after Wald to identify the relevant variables.

To verify the blood values for possible correlations with rate of decay and the ALSFRS-R score at study entry, we carried out linear regressions. We calculated univariate models to quantify an influence of the different blood parameters on the dependent variable. Afterwards, we controlled the model for gender, age at onset, diagnostic delay, region of onset, UMN and LMN predominance. The same procedure was used for the follow-up subgroup with the progression rate as dependent variable.

Since only two centers (Hanover and Lisbon) provided data on survival, we analyzed this subgroup separately regarding the influence of the variables on survival. We created Kaplan-Meier diagrams for each of the blood parameters to compare groups defined by blood values above or below the reference value regarding their survival time since diagnosis. For further survival analysis we established Cox-regression models with time between diagnosis and death or censorship as dependent variable. Patients without a date of death were censored at the date they were contacted the last time. Once more, we included each variable separately into univariate models and controlled afterwards for aforementioned variables in multivariate analyses.

Because of the inflation of the alpha error due to multiple testing in the same sample, the collected *p*-values were assessed descriptively. The term “significant” was avoided.

Data analysis was carried out with the Statistical Package for the Social Science Version 24 (SPSS, Chicago, IL).

## Results

### Patient's characteristics

41.6% of our patients were females and the mean age at diagnosis was 61.07 years. While the main part presented with limb onset (70.5%), a quarter showed bulbar symptoms at first (25.7%). Only few patients had thoracic symptoms (abdominal/respiratory musculature, 2.6%) or dyscognition (1.1%) as first disease manifestation. On average the diagnosis was delayed by 17.51 months after disease onset. While most patients initially presented with both LMN/UMN signs, 11.6% had pure LMN involvement (pure lower motor neuron disease) and 1.4% pure UMN involvement (primary lateral sclerosis). In the group with combined LMN and UMN signs LMN involvement was predominant in 74.5% and 27.6% had UMN predominance. Few patients in whom no predominance of a motoneuron could be detected were included in both groups.

In Tables 1.1, 1.2 the characteristics of different blood parameter groups are described. It is visible that there are no relevant differences in the composition of basic variables between these groups.

### Correlation of blood parameters with disease characteristics

First, we intended to identify if certain blood parameters were associated with particular disease characteristics. Individual results including *p*-value and CI 95% of the univariate and multivariate calculations for all blood values can be found in Supplementary Tables 1–7. Relevant correlations were finally determined by means of backward selection as summarized in Tables 2.1, 2.2.

**Table 2.1:** Correlation of disease characteristics and blood parameters.

**Creatine kinase** **↑**	**Albumin** **↓**	**Creatinine** **↑**
Male gender	No LMN predominance	Male gender
Age at diagnosis ↓	Diagnostic delay ↑	Age at diagnosis ↑
LMN predominance		No LMN predominance
		No lower limb involvement
Lower limb involvement		Physical exercise

**Table 2.2:** Correlation of disease characteristics and blood parameters.

**Total cholesterol** **↑**	**HDL↑**	**LDL↑**	**Triglyceride↑**
Female gender	Female gender	Female gender	Male gender
Thoracic onset*	Age at diagnosis ↑	Physical exercise	Statin intake
Physical exercise	Physical exercise		LMN predominance
			Lower limb involvement

* *In comparison to limb onset*.

A higher CK level was associated with male gender, lower age at diagnosis, LMN predominance and lower limb involvement (Table 2.1 and Supplementary Table 1).

Lower ALB levels were observed in patients without LMN predominance and a longer diagnostic delay. Females tended to have lower ALB levels, but this finding did not reach a *p*-value <0.05 (Table 2.1 and Supplementary Table 2).

Male gender, older age at diagnosis, no LMN predominance or no lower limb involvement and physical exercise were associated with higher CREA values (Table 2.1, Supplementary Table 3).

The regression model for TC levels revealed female gender, thoracic onset (in comparison to limb onset) and physical exercise to be linked with higher levels (Table 2.1 and Supplementary Supplementary Table 4).

Higher HDL levels were associated with female gender, older age at onset and physical exercise (Table 2.1 and Supplementary Table 5).

LDL levels were higher in female patients and those doing physical exercise (Table 2.1 and Supplementary Table 6).

Lastly, higher TG levels were associated with male gender, statin intake, LMN predominance and involvement of the limbs (Table 2.1 and Supplementary Table 7).

None of the blood values correlated with the area of living in the last 5 years or before.

### Correlation of blood parameters with severity of disease and disease progression

Lower CK levels were associated with faster rate of decay in the univariate model [*p*-value 0.006, *95%CI* (−0.001, 0.000)]. Nevertheless, the CI contained a zero as boundary and this effect vanished after controlling the model for basic variables mentioned above. Higher TG levels were by trend associated with a faster rate of decay in the univariate [*p*-value 0.092, *95%CI* (0.000, 0.002)], and multivariate analysis controlled for basic variables [*p*-value 0.08, *95%CI* (0.000, 0.0002)] (Table 3). Interestingly, the subgroup analysis of the individual countries gave a relevant positive correlation between TG and the rate of decay for patients from Germany and Portugal (Supplementary Tables 8A,B). No association between ALB, CREA, TC, HDL cholesterol, and LDL cholesterol and rate of decay could be detected.

**Table 3:** Linear regression models for rate of decay and blood parameters.

	**Univariate analysis**			Multivariate analysis*			**Rate of decay** **↑**
**Variable**	*****n*****	*****p***** **-value**	**95% CI**	*****n*****	*****p-***** **value**	**95% CI**	
CK	802	0.006	(−0.001, 0.000)	747	0.285	(0.000, 0.000)	CK↓
Albumin	518	0.547	(−0.238, 0.126)	482	0.299	(−0.295, 0.091)	
Creatinine	973	0.784	(−0.182, 0.254)	904	0.939	(−0.216, 0.233)	
Total cholesterol	690	0.604	(−0.001, 0.002)	651	0.857	(−0.001, 0.001)	
HDL	607	0.636	(−0.005, 0.003)	564	0.262	(−0.006, 0.002)	
LDL	603	0.28	(−0.001, 0.003)	563	0.771	(−0.001, 0.002)	
Triglyceride	656	0.092	(0.000, 0.002)	612	0.08	(0.000, 0.002)	

* *Controlled for gender, age at diagnosis, diagnostic delay, region of onset, UMN and LMN involvement.*

*Bold values show p-values < 0.05*.

Also, there was no connection between any blood value and the progression rate in our analysis of patients with follow-up data. Lower ALB levels in the univariate model tended to be associated with a faster progression rate [*p*-value 0.074, *95%CI* (−0.83, 0.039)] (Table 4).

**Table 4:** Linear regression models for progression rate and blood parameters.

	**Univariate analysis**			Multivariate analysis*			**Progression rate** **↑**
**Variable**	*****n*****	*****p***** **-value**	**95% CI**	*****n*****	*****p-***** **value**	**95% CI**	
CK	213	0.393	(−0.001, 0.000)	225	0.877	(−0.001, 0.001)	
Albumin	151	0.074	(−0.83, 0.039)	147	0.13	(−0.82, 0.108)	
Creatinine	262	0.158	(−0.183, 1.122)	254	0.456	(−0.457, 1.016)	
Total cholesterol	190	0.161	(−0.007, 0.001)	183	0.6	(−0.005, 0.003)	
HDL	143	0.847	(−0.01, 0.008)	137	0.921	(−0.01, 0.009)	
LDL	148	0.844	(−0.005 0.004)	142	0.932	(−0.005, 0.005)	
Triglyceride	169	0.331	(−0.005, 0.002)	163	0.541	(−0.004 0.002)	

* *Controlled for gender, age at diagnosis, diagnostic delay, region of onset, UMN and LMN involvement*.

Regarding the functional status at study entry representing severity of disease, lower CK [*p*-value 0.001, *95%CI* (0.002, 0.007)], ALB [*p*-value <0.001, *95%CI* (3.964, 7.035)], CREA [*p*-value <0.001, *95%CI* (3.664, 7.605)], and HDL [*p*-value 0.044, *95%CI* (0.001, 0.079)] levels were associated with lower ALSFRS-R scores at study entry in the univariate and multivariate analysis. Higher TG levels tended to show a similar connection in the univariate analysis (Table 5).

**Table 5:** Linear regression models for ALSFRSR at study entry and blood parameters.

	**Univariate analysis**			Multivariate analysis*			**ALSFRSR** **↓**
**Variable**	*****n*****	*****p***** **-value**	**95% CI**	*****n*****	*****p-***** **value**	**95% CI**	
CK	803	<0.001	(0.003, 0.008)	747	0.001	(0.002, 0.007)	CK↓
Albumin	518	<0.001	(4.073, 6.947)	482	<0.001	(3.964, 7.035)	Alb↓
Creatinine	974	<0.001	(3.965, 7.68)	904	<0.001	(3.664, 7.605)	Crea↓
Total cholesterol	690	0.385	(−0.007, 0.018)	651	0.371	(−0.007, 0.019)	
HDL	607	0.044	(0.001, 0.075)	564	0.044	(0.001, 0.079)	HDL↓
LDL	603	0.395	(−0.023, 0.009)	563	0.462	(−0.022, 0.01)	
Triglyceride	656	0.097	(−0.017, 0.001)	612	0.061	(−0.019, 0.000)	

* *Controlled for gender, age at diagnosis, diagnostic delay, region of onset, UMN and LMN involvement.*

*Bold values show p-values < 0.05*.

### Correlation of blood parameters with survival

To determine if any blood value is associated with survival we performed Cox-regression and Kaplan-Meier analyses.

**Figure 2:**
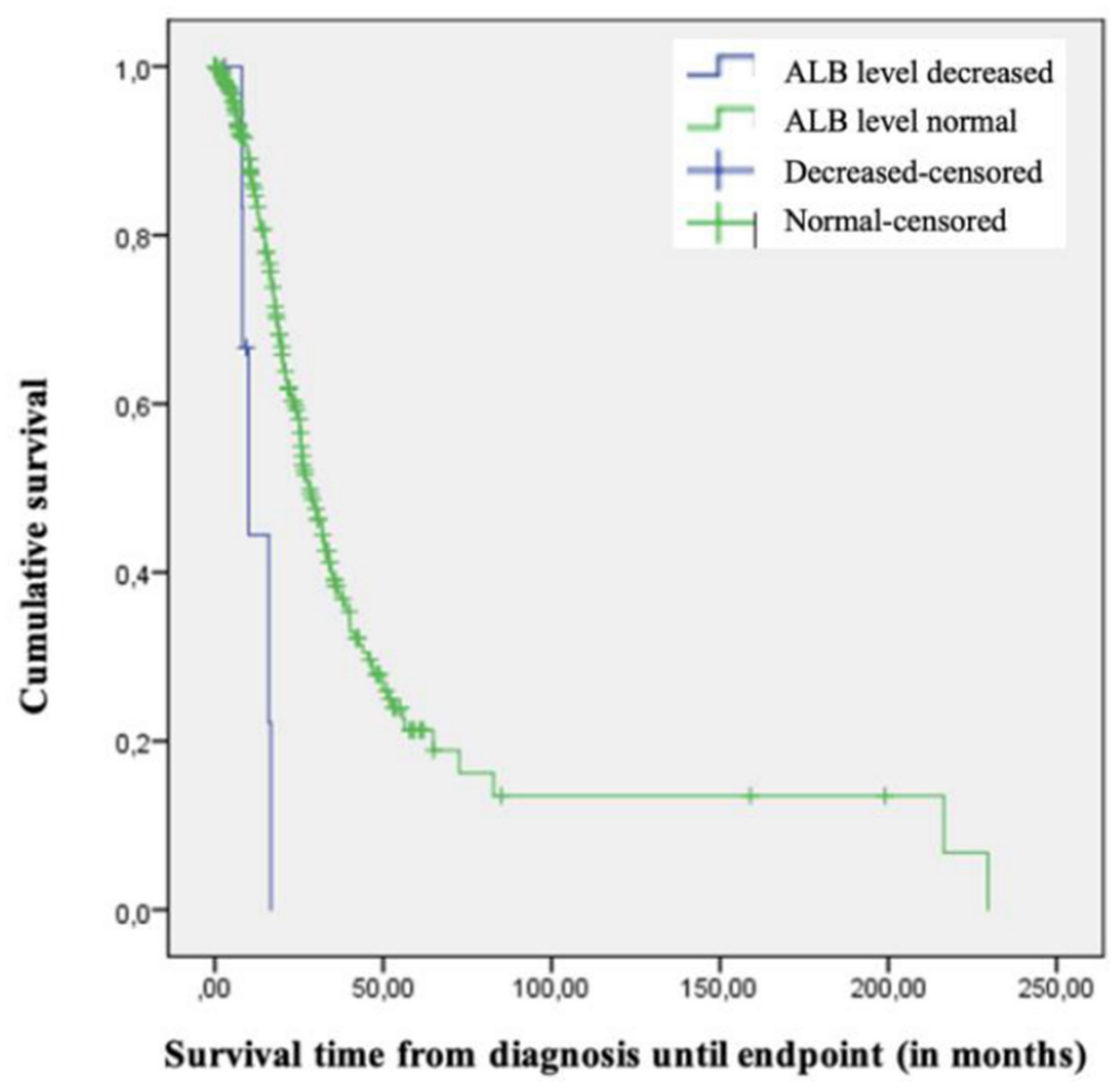
Kaplan-Meier-Diagram of survival time since diagnosis based on albumin level.

The Kaplan-Meier survival diagrams revealed that patients with increased CREA or TC levels tended to have a longer survival time, though these observations did not reach significance *(log-rank test 0.08 resp. 0.057 for TC*). On the other hand, lower ALB levels were associated with shorter survival (*log-rank test* <0.001) (Figure 2). Yet it must be mentioned that there were only 7 patients with decreased ALB levels. The phenotype of these 7 patients was characterized by a spinal onset in 5 patients and a bulbar onset in 2 patients. The 7 patients with low albumin were older with an average age of 74 years at onset and had a shorter diagnostic delay of 12.85 months.

In the univariate Cox-regression models lower CK [*p*-value 0.043, *HR* 0.999, *95%CI* (0.999, 1.0)], lower TC [*p*-value 0.026, *HR* 0.996 *95%CI* (0.992, 1.0)] and lower LDL levels [*p*-value 0.025, *HR* 0.995, *95%CI* (0.99, 0.999)] were associated with a higher risk of death. These associations vanished after controlling the model for basic variables. Even though, the univariate model did not show a connection between CREA and risk of death, after controlling this model for basic variables lower CREA values were associated with a higher risk of death [*p*-value 0.003, *HR* 0.337, *95%CI* (0.166, 0.685)] (Table 6).

**Table 6:** Cox regression models for time from diagnosis until death/censorship.

	**Univariate analysis**			**Multivariate analysis**			**Risk of death** **↑**
**Variable**	***n***	***p*-value**	**HR (95% CI)**	***n***	***p*-value**	**HR (95% CI)**	
CK	436	0.043	0.999 (0.999, 1.0)	429	0.824	1.0 (0.999, 1.001)	
Albumin	291	0.496	0.876 (0.598, 1.284)	286	0.286	0.987 (0.648, 1.503)	
Creatinine	524	0.421	0.784 (0.433, 1.419)	515	0.003	0.337 (0.166, 0.685)	Crea ↓
Total cholesterol	388	0.026	0.996 (0.992, 1.0)	382	0.224	0.998 (0.994, 1.001)	
HDL	307	0.956	1.0 (0.991, 1.009)	301	0.652	0.998 (0.998, 1.008)	
LDL	313	0.025	0.995 (0.99, 0.999)	307	0.155	0.996 (0.991, 1.001)	
Triglyceride	353	0.979	1.0 (0.998, 1.002)	347	0.818	1.0 (0.998, 1.003)	

*Controlled for gender, age at onset, diagnostic delay, region of onset, UMN, and LMN.*

*Bold values show p-values < 0.05*.

## Discussion

In a large international population of ALS patients, we investigated various blood values that could be promising as possible biomarkers. For the individual parameters the results are discussed below in the context of the previous literature.

### Creatine kinase (CK)

Many studies examined CK as potential biomarker in the past, but the results are rather conflicting. Our analysis showed that high CK values occur when large muscle masses are affected. Younger men with LMN predominance in the legs are most likely to have high CK levels.

Other researchers also found a correlation of CK with LMN loss and muscle atrophy due to denervation (9–11, 13). Intact innervation seems to be crucial to regulate the CK levels and denervation results in instability of the cell membrane, thus releasing enzymes into the bloodstream (47, 48). In consequence, increased CK levels could reflect CK leakage from denervated or atrophied muscles, while the extent of this increase might depend on the amount of afflicted musculature. The potential of CK as biomarker for the extent of involved muscle mass and severity, progression or prognosis has therefore been suggested before (5). However, it does not appear that a high CK level reflects strong disease activity or is associated with poor functional status or rapid disease progression. Our results rather show a correlation of high CK values with a better ALSFRS-R score as previously described (13). Others failed to show an association of CK levels with disease severity (8, 9, 16). Our findings could be explained by higher baseline muscle mass and therefore better functional performance in patients with higher CK levels. In addition, high CK levels seem to be particularly eminent at disease onset when substantial muscle mass is still present. Decrease of CK levels in correlation with increasing muscle atrophy has indeed been observed in earlier studies (13, 16), even though not in all of them (6). Rafiq et al. found increasing levels between 0 and 3 months, decreasing levels between 3 and 12 months and constant levels between 12 and 18 months after disease onset (9). Additionally, CK levels can be reduced by an increase in reactive oxygen species and mitochondrial dysfunction, which both have been identified as important ALS mechanisms (49) and therefore lower CK levels might reflect a later disease stage. Since CK is an enzyme involved in the muscle's energy metabolism (19), some researchers also assumed that upregulation improves muscle energy and function. This is supported by the finding that patients with higher CK levels tend to show slower progression (20, 33) and that CK declines faster in rapidly progressive disease (50). We found an association between rate of decay and CK levels in the linear univariate model, but the result was not reproducible in the multivariate model including basic variables. This suggests that the relationship of CK and rate of decay is not independent of variables such as age and gender.

Some studies found higher CK levels correlated with longer survival (9, 10, 16, 23) and thus suggested that CK might have a protective function. The results of Gibson et al. were the only ones claiming worse survival in patients with higher baseline CK levels (12), while others including us failed to find an association between survival and CK level at all (4, 6, 21, 22). A recent meta-analysis negated an influence of CK on survival as well (15). As an increase in CK can arise either from release from denervated muscles or from compensatory upregulation to improve muscular energy metabolism, future studies should take into consideration that CK has a short half lifetime of 22 h (9) and levels are influenced by age and gender and also by physical activity or cramps. Repeated measurements under controlled conditions might finally set its role in the prediction of disease severity, progression, or prognosis.

### Albumin

We found lower ALB values at diagnosis to be associated with a lower ALSFRS-R score at study entry and a longer diagnostic delay. Other studies have already shown similar results (26, 51). Albumin is well-known as a marker for inflammation, with lower levels being associated with higher inflammatory activity and correlated with inflammatory markers (26). Furthermore, it has antioxidative properties, so higher levels might provide protection against oxidative stress in ALS, while oxidative stress can also distort albumin functionality (52). Since neuroinflammation and oxidative stress are mechanisms involved in the pathogenesis of ALS (53–55), the level of disease activity might be reflected by ALB levels. This could explain the potential of ALB as a marker of severity or progression, since a higher disease activity—and therefore possibly worse functional performance—goes along with more severe inflammation, which can decrease ALB and lead to less protection against oxidative stress. Albumin is also known as a marker for the nutritional status and since dysphagia is a common phenomenon in ALS due to bulbar degeneration, this feature might contribute to the explanation of our findings as well. This approach is supported by a study of Chelstowska et al., where a correlation between ALB and ALSFRS-R score was apparent even after exclusion of patients with inflammatory markers (51). Decreasing ALB levels were found in patients with faster progression, whereas ALB levels increased in those with slower progression (50). Additionally, higher ALB levels were associated with longer survival or lower risk of death in recent studies (26, 27), even though some other researchers failed to confirm this (21, 50). In patients with percutaneous endoscopic gastrostomy (PEG) those with normal and increasing ALB levels had the longest survival time (28). Especially in patients with bulbar symptoms, early implementation of PEG can be necessary to counteract malnutrition and weight loss due to dysphagia. Lopez-Gomez et al. could even show prolonged survival in ALS patients with PEG (56). Our Kaplan-Meier diagrams actually did show this tendency too, yet the group of patients with low ALB only contained 7 patients. It needs to be mentioned that some data was missing regarding survival time, so our findings must be interpreted with caution. Overall, the current evidence suggests that low ALB levels are more likely to be associated with poor functional status and prognosis. Some authors only focused on patients with PEG though ALB might also be affected by nutritional status in general, changes in metabolism and neuroinflammation and thus important in all ALS patients. Future studies should concentrate on the longitudinal decline in ALB to better evaluate its potential as biomarker.

### Creatinine

In our cohort, CREA was higher in male patients, patients without LMN predominance or lower limb involvement and in those being physically active. This can be explained by the fact that in comparison these patients might have more muscle mass than patients with LMN predominance or lower limb involvement or physically inactive patients. Older patients had higher CREA levels than younger ones which might be ascribed to decreasing renal function. Lower CREA levels at diagnosis were associated with lower ALSFRS-R scores at study entry. This observation has been made in other studies as well (22, 26, 36, 51) and some of them were also able to show an inverse correlation between CREA and the ALSFRS-R decline (22, 31–33, 57), which our data did not show. CREA values reflect the amount of muscular mass (29). Not only are CREA levels lower in ALS patients in comparison with healthy controls (32, 36), but low values also precede the diagnosis about 2 years in advance (58), which might reflect disease activity even before a diagnosis can be made. Patin et al. did not find low CREA levels in every patient with atrophy (34), but a decrease over the disease course could be observed (22, 34). Additionally, low levels are associated with severe muscle impairment in ALS (31). CREA was not significantly associated with survival in our cohort in the univariate analysis. However, CREA depends on different variables such as renal function, age, gender, protein intake and general physical activity (59). After we adjusted the model for age at diagnosis, gender, and other disease characteristics, we detected a higher risk of death in patients with lower CREA levels. This observation is supported by several other research groups (22, 23, 26, 27, 30, 31, 57).

Creatinine is a breakdown product of creatine phosphate and reflects the creatine pool as well as the impaired uptake of creatine into muscle cells. Low CREA levels might therefore be associated with an aggravation of the disease progression and could indicate a shorter survival, as creatine stabilizes mitochondrial structures and their functionality and provides antioxidative functions (19). Creatinine is an easily accessible blood parameter, which makes it a promising candidate for a more objective estimation and monitoring of the functional status in addition to the ALSFRS-R. Using CREA as surrogate endpoint in clinical studies could decrease their costs and increase their power (60). Future studies should concentrate on the slope of CREA, which seems to decrease, and their models need to be adjusted for variables that influence CREA such as renal function, age, and BMI, since for example BMI is an independent indicator for survival (37). An influence of those parameters on the correlation of CREA and ALSFRS-R needs to be ruled out in order to establish CREA as biomarker for disease severity, progression, or prognosis.

### Lipid values

Blood lipids have been extensively studied as ALS biomarkers in the past decade. The central nervous system contains high amounts of lipids, which are crucial for different mechanisms such as signal transduction, neuronal structure, and steroid hormone synthesis (61, 62). They also provide energy, especially if glucose is rare or glucose metabolism is impaired (62). ALS patients indeed show an abnormal carbohydrate metabolism (63) and a metabolic shift from glucose to fatty acids as energy substrate. This might be due to degeneration of mainly glycolytic muscle fibers, whereas oxidative, fat-requiring muscle fibers are resistant. Therefore, lipids are thought to play an important role in ALS pathogenesis and progression (64, 65). Furthermore, ALS patients have a higher resting energy expenditure, which increases in the course of disease (18, 66), making lipids even more crucial for energy supply. Hypermetabolic patients tend to have worse prognosis compared to patients with normal metabolism (38). ALS patients are also predestined for malnutrition due to dysphagia and therefore in need of more energy. A higher BMI is known to be associated with longer survival (37). High-fat diets were protective in mouse models (67, 68) and a clinical trial assessing a high-caloric fatty diet did not reach its primary endpoint but showed benefit in fast progressing patients (69). Overall, the potential of blood lipids for predicting disease risk, prognosis or progression remains inconclusive. In our analysis higher TC, HDL, and LDL values were associated with female gender and higher TG values with male gender. Why elevated LDL cholesterol was associated with increased physical activity remains unclear. Physical exercise can lead to an increase in HDL cholesterol, but does not affect LDL cholesterol (70, 71). One explanation could be that patients with elevated LDL cholesterol were advised to increase physical activity. Only HDL cholesterol revealed a relevant correlation with the ALSFRS-R score, where higher HDL levels were associated with better functional status. In contrast, higher TG levels were by trend associated with lower ALSFRS-R scores and higher rates of decay, yet these results did not reach significance when analyzing the whole cohort. In a subgroup analysis of the individual countries, this relationship was interestingly shown to be relevant for Germany and Portugal. This could indicate that different lifestyle and dietary habits as well as different comorbidity profiles in the diverse countries could play a role in the correlations with disease progression.

Regarding survival analyses, higher TC and LDL levels were linked with lower risk of death in the univariate analysis, but the association vanished after controlling for basic variables.

There is an ongoing debate, whether dyslipidemia is a risk or protective factor, as well as if it influences the progression positively, negatively, or not at all. Dupuis et al. found higher lipid levels in ALS patients compared to healthy controls (72), even though others could only confirm this observation in females (32, 72, 73). Higher cholesterol intake as well as higher TC or LDL levels and LDL/HDL ratios before disease onset have been shown to be associated with higher ALS incidences or risk of disease (74–76). Kim et al. on the other hand showed hypolipidemia in male ALS transgenic mice compared to wild type controls (77), and Sutedja et al. found ALS patients to have a more favorable cardiovascular risk factor profile (low TC and LDL, high HDL) and lower BMI (78), which is supported again by different other studies (79–81). Further studies including a recent meta-analysis failed to confirm a difference in any of the lipid values between ALS patients and healthy controls (74, 82, 83). Regarding the potential for predicting disease severity and influencing progression, lower lipids (TC and LDL) have been shown to be associated with lower FVC or more rapid decline (32, 78, 82). Some studies revealed a positive correlation of the ALSFRS-R score with LDL, HDL, and TG levels as well as inversely with TC levels (84), which at least concerning HDL was supported by our study. Furthermore, in one of these analyses, faster decline of ALSFRS-R score was associated with lower TC and LDL levels (32). Moreover, some researchers found higher lipid levels being associated with longer survival (21, 61, 72, 73, 78, 83, 85–87), but this association mostly vanished after controlling the model for different confounders such as BMI, age at onset or diagnosis, site of onset or FVC as seen in our study as well regarding LDL and TC. Other studies failed to show a correlation between lipid levels and ALSFRS-R score, disease progression or survival at all (21, 37, 81–83). Patin et al. even showed higher LDL/HDL ratios being associated with shorter survival (34), but again this effect vanished after adjustment for BMI. The above-mentioned meta-analysis failed to show any correlation between lipid levels and mortality (88). A possible explanation for these conflicting results might be the fact that circulating lipids are influenced by many different factors including genetics, metabolism, nutrition, gender, lifestyle and even more important the time of day as well as the time after the last food intake (89). Moreover, ALS is a very heterogeneous disease. Lipids might play a role in ALS risk and progression but more differentiated studies of ALS subgroups with standardized analysis programs and rigorous adjustment for confounding factors are needed to identify which blood lipid level may actually have prognostic value and which patients might profit from higher lipid levels the most.

### Limitations

Our study has some limitations, which must be kept in mind while interpreting the results. Because of the multi-center design, the data sets were not complete with all blood values for each patient. We addressed this problem by analyzing different patient groups according to blood parameters and described them separately to show that they did not differ much in their baseline characteristics. Although we included some factors that may influence the blood values studied in our multivariate analyses, many other patient characteristics, such as present comorbidities or eating habits, could still be relevant and were not considered in the present study. Not all patients were available for follow up information. Those were censored at the last date we heard from them, which might blur the survival analyses. Furthermore, the follow-up data was not collected in person but by telephone or e-mail and my therefore sometimes lack accuracy. Additionally, the blood values were analyzed in different laboratories, which might lead to bias since the measuring methods might differ slightly. Recall bias is a common problem in our study as well since many questions in our questionnaire regarded events in the past.

## Conclusion

Our study indicates that CK, ALB, CREA, and HDL levels at diagnosis are related to the functional status and can potentially reflect disease severity. Thus, they might be promising candidates for biomarkers as supported by previous study results. Our univariate and multivariate analyses highlight that it is crucial not to consider isolated correlations of individual blood values with disease characteristics. These parameters are not disease-specific and easily influenced by different factors as age, gender, physical activity, and several conditions around the time of blood sampling. Future studies need to control their data for those influencing factors and should assess blood levels longitudinally to give more insight into their potential as biomarker. Most likely repeated examination of a particular combination of blood values will have a better potential for predicting ALS severity and progression.

## Data availability statement

The original contributions presented in the study are included in the article/supplementary material, further inquiries can be directed to the corresponding author/s.

## Ethics statement

The studies involving human participants were reviewed and approved by the Ethical Committees of all participating centers. Theses were: Ethics Committee, Medical Centre of Faculty of Medicine, in Lisbon, Portugal; Ethics Committee, Hannover Medical School, Germany; Ethics Committee, Medical University of Warsaw, Warsaw, Poland; Ethics Committee , Akdeniz University, Antalya, Turkey; and the Ethics Committee, University Jena, Germany. The patients/participants provided their written informed consent to participate in this study.

## Author contributions

SK and NH designed the study, collected the data, analyzed the data, interpreted the data, and drafted the article. MK-K, MG, JG, MC, HU, RD, and SP contributed to acquisition and analysis of data and critically revised the article for important intellectual content. All authors contributed to the article and approved the submitted version.

## Funding

The study was funded by the European Union (ONWebDUALS project, JNPD 01ED1511B).

## Conflict of interest

The authors declare that the research was conducted in the absence of any commercial or financial relationships that could be construed as a potential conflict of interest.

## Publisher's note

All claims expressed in this article are solely those of the authors and do not necessarily represent those of their affiliated organizations, or those of the publisher, the editors and the reviewers. Any product that may be evaluated in this article, or claim that may be made by its manufacturer, is not guaranteed or endorsed by the publisher.

## Abbreviations

ALB: albumin
ALSFRS-R: ALS functional rating scale-revised
CK: creatine kinase
CREA: creatinine
HDL: high-density lipoprotein cholesterol
LDL: low-density lipoprotein cholesterol
LMN: lower motor neuron
TC: total cholesterol
TG: triglyceride
UMN: upper motor neuron.
