# Supplementary material for: Analysis of routine blood parameters in patients with amyotrophic lateral sclerosis and evaluation of a possible correlation with disease progression—a multicenter study
Source: Front Neurol. 2022 Jul 27;13:940375. doi: 10.3389/fneur.2022.940375 (PMC9364810; doi:10.3389/fneur.2022.940375)
Supplement: Supplementary file 1 [file Table_1.DOCX]

Supplemental Table 1 Correlation of disease characteristics/living conditions and CK level

|  | Univariat analysis | | Multivariate analysis (n = 745) | | Multivariate analysis backward selection | |
| --- | --- | --- | --- | --- | --- | --- |
|  | *p* value | 95% CI | *p* value | 95% CI | *p* value | 95% CI |
| Basics | | | | | | |
| Gender  (n = 816) | **<0.001** | (74.579, 138.212) | **<0.001** | (51.883, 122.341) | **<0.001** | (63.866, 130.335) |
| Age at diagnosis (n = 791) | **<0.001** | (-3.877, -1.419) | **0.004** | (-3.243, -0.606) | **<0.001** | (-3.45, -0.997) |
| Statin intake (n = 810) | **0.022** | (-97.223, -7.445) | 0.516 | (-63.09, 31.705) |  |  |
| Disease characteristics | | | | | | |
| Limb onset  (n = 816) | Reference |  | Reference |  |  |  |
| Bulbar onset (n = 816) | **<0.001** | (-139.224, -65.766) | 0.213 | (-75.966, 16.93) |  |  |
| Thoracic onset (n = 816) | 0.839 | (0.119.372, 97.03) | 0.833 | (-97.588, 121.06) |  |  |
| Dyscognition onset (n = 816) | 0.248 | (-322.684, 83.497) | 0.841 | (-253.745, 206.806) |  |  |
| Predominant UMN (n = 780) | **<0.001** | (-110.83, -36.702) | 0.763 | (-54.163, 73.869) |  |  |
| Predominant LMN (n = 780) | **<0.001** | (52.006, 133.998) | **0.015** | (16.756, 158.46) | **<0.001** | (47.745, 128.612) |
| Upper limb (n = 816) | 0.699 | (-40.054, 26.878) |  |  |  |  |
| Lower limb (n = 816) | **<0.001** | (46.547, 111.395) | **0.001** | (24.885, 100.488) | **<0.001** | (43.211, 109.451) |
| Diagnostic delay (n = 790) | **0.043** | (0.024, 1.413) | 0.313 | (-0.337, 1.049) |  |  |
| Health-related behavior | | | | | | |
| Smoking (n = 809) | **0.011** | (9.514, 74.174) | 0.68 | (-27.213, 41.681) |  |  |
| PE (n = 806) | **0.002** | (18.042, 82.665) | 0.223 | (-12.894, 55.277) |  |  |
| Living conditions |  |  |  |  |  |  |
| Living area >5years (rural/urban) (n = 746) | 0.384 | (-54.901, 21.15) |  |  |  |  |
| Living area in the last 5 years (rural/urban) (n = 782) | 0.255 | (-57.39, 15.242) |  |  |  |  |
