# Supplementary material for: Analysis of routine blood parameters in patients with amyotrophic lateral sclerosis and evaluation of a possible correlation with disease progression—a multicenter study
Source: Front Neurol. 2022 Jul 27;13:940375. doi: 10.3389/fneur.2022.940375 (PMC9364810; doi:10.3389/fneur.2022.940375)
Supplement: Supplementary file 2 [file Table_2.DOCX]

Supplemental Table 2 Correlation of disease characteristics/living conditions and albumin level

|  | Univariat analysis | | Multivariate analysis (n = 458) | | Multivariate analysis backward selection | |
| --- | --- | --- | --- | --- | --- | --- |
|  | *p* value | 95% CI | *p* value | 95% CI | *p* value | 95% CI |
| Basics | | | | | | |
| Gender  (n = 521) | **0.173** | (-0.028, 0.153) | 0.121 | (-0.019, 0.163) | 0.081 | (-0.01, 0.171) |
| Age at diagnosis (n = 502) | **0.027** | (-0.007, 0.000) | 0.18 | (-0.006, 0.001) |  |  |
| Disease characteristics | | | | | | |
| Limb onset  (n = 521) | Reference |  |  |  |  |  |
| Bulbar onset (n = 521) | 0.857 | (-0.115, 0.096) |  |  |  |  |
| Thoracic onset (n = 521) | 0.962 | (-0.286, 0.272) |  |  |  |  |
| Dyscognition  (n = 521) | 0.989 | (-0.458, 0.464) |  |  |  |  |
| Predominant UMN (n = 500) | **0.117** | (-0.169, 0.019) | 0.855 | (-0.187, 0.155) |  |  |
| Predominant LMN (n = 500) | **0.042** | (0.004, 0.214) | 0.462 | (-0.119, 0.262) | **0.036** | (0.008, 0.22) |
| Upper limb (n = 521) | **0.108** | (-0.017, 0.168) | 0.264 | (-0.041, 0.15) |  |  |
| Lower limb (n = 521) | 0.238 | (-0.147, 0.037) |  |  |  |  |
| Diagnostic delay (n = 502) | **0.037** | (-0.004, 0.000) | **0.045** | (-0.004, 0.000) | **0.039** | (-0.004, 0.000) |
| Health-related behavior | | | | | | |
| Smoking (n = 518) | 0.271 | (-0.04, 0.142) |  |  |  |  |
| PE (n = 517) | 0.336 | (-0.046, 0.134) |  |  |  |  |
| Living conditions |  |  |  |  |  |  |
| Living area >5years (rural/urban) (n = 460) | 0.664 | (-0.128, 0.08) |  |  |  |  |
| Living area in the last 5 years (rural/urban) (n = 460) | **0.047** | (-0.194, -0.001) | 0.145 | (-0.167, 0.025) |  |  |
