# Supplementary material for: Analysis of routine blood parameters in patients with amyotrophic lateral sclerosis and evaluation of a possible correlation with disease progression—a multicenter study
Source: Front Neurol. 2022 Jul 27;13:940375. doi: 10.3389/fneur.2022.940375 (PMC9364810; doi:10.3389/fneur.2022.940375)
Supplement: Supplementary file 3 [file Table_3.DOCX]

Supplemental Table 3 Correlation of disease characteristics/living conditions and creatinine level

|  | Univariat analysis | | Multivariate analysis (n = 905) | | Multivariate analysis backward selection | |
| --- | --- | --- | --- | --- | --- | --- |
|  | *p* value | 95% CI | *p* value | 95% CI | *p* value | 95% CI |
| Basics | | | | | | |
| Gender  (n = 990) | **<0.001** | (0.105, 0.176) | **<0.001** | (0.115, 0.192) | **<0.001** | (0.109, 0.182) |
| Age at diagnosis (n = 964) | **<0.001** | (0.003, 0.006) | **<0.001** | (0.003, 0.006) | **<0.001** | (0.003, 0.006) |
| Disease characteristics | | | | | | |
| Limb onset  (n = 990) | Reference |  | Reference |  |  |  |
| Bulbar onset (n = 990) | **0.015** | (0.01, 0.092) | 0.168 | (-0.015, 0.086) |  |  |
| Thoracic onset (n = 990) | 0.582 | (-0.078, 0.139) | 0.821 | (-0.118, 0.094) |  |  |
| Dyscognition  (n = 990) | 0.923 | (-0.162, 0.179) | 0.517 | (-0.262, 0.132) |  |  |
| Predominant UMN (n = 941) | 0.384 | (-0.021, 0.06) |  |  |  |  |
| Predominant LMN (n = 941) | **0.093** | (-0.083, 0.006) | **0.043** | (-0.093, -0.002) | **0.014** | (-0.097, -0.011) |
| Upper limb (n = 990) | 0.878 | (-0.034, 0.04) |  |  |  |  |
| Lower limb (n = 990) | **0.026** | (-0.079, -0.005) | 0.079 | (-0.079, 0.004) | **0.01** | (-0.086, -0.012) |
| Diagnostic delay (n = 963) | 0.482 | (-0.001, 0.000) |  |  |  |  |
| Health-related behavior | | | | | | |
| Smoking (n = 981) | **0.036** | (0.003, 0.074) | 0.514 | (-0.051, 0.025) |  |  |
| PE (n = 978) | **<0.001** | (0.048, 0.119) | **<0.001** | (0.034, 0.108) | **<0.001** | (0.034, 0.107) |
| Living conditions |  |  |  |  |  |  |
| Living area >5years (rural/urban) (n = 903) | 0.898 | (-0.039, 0.045) |  |  |  |  |
| Living area in the last 5 years (rural/urban) (n = 903) | 0.542 | (-0.053, 0.028) |  |  |  |  |
