# Supplementary material for: Analysis of routine blood parameters in patients with amyotrophic lateral sclerosis and evaluation of a possible correlation with disease progression—a multicenter study
Source: Front Neurol. 2022 Jul 27;13:940375. doi: 10.3389/fneur.2022.940375 (PMC9364810; doi:10.3389/fneur.2022.940375)
Supplement: Supplementary file 4 [file Table_4.DOCX]

Supplemental Table 4 Correlation of disease characteristics/living conditions and total cholesterol level

|  | Univariat analysis | | Multivariate analysis (n = 618) | | Multivariate analysis backward selection | |
| --- | --- | --- | --- | --- | --- | --- |
|  | *p* value | 95% CI | *p* value | 95% CI | *p* value | 95% CI |
| Basics | | | | | | |
| Gender  (n = 703) | **<0.001** | (-33.086, -7.511) | **<0.001** | (-25.347, -9.446) | **<0.001** | (-25.3, -9.473) |
| Age at diagnosis (n = 687) | 0.752 | (-0.24, 0.332) |  |  |  |  |
| Statin intake (n = 700) | **0.094** | (-18.095, 1.432) | 0.298 | (-15.493, 4.76) |  |  |
| Disease characteristics | | | | | | |
| Limb onset  (n = 703) | Reference |  | Reference |  | Reference |  |
| Bulbar onset (n = 703) | 0.618 | (-10.466, 6.229) | 0.492 | (-12.688, 6.111) | 0.315 | (-13.607, 4.388) |
| Thoracic onset (n = 703) | **0.022** | (4.488, 56.769) | **0.028** | (3.298, 57.219) | **0.024** | (4.017, 57.534) |
| Dyscognition (n = 703) | 0.26 | (-62.353, 16.886) | 0.549 | (-65.458, 34.816) | 0.218 | (-77.762, 17.805) |
| Predominant UMN (n = 675) | **0.17** | (-14.041, 2.477) | 0.721 | (-18.881, 13.076) |  |  |
| Predominant LMN (n = 675) | **0.168** | (-2.8, 16.045) | 0.733 | (-14.699, 20.89) |  |  |
| Upper limb (n = 703) | 0.421 | (-10.616, 4.44) |  |  |  |  |
| Lower limb (n = 703) | 0.884 | (-8.11, 6.987) |  |  |  |  |
| Diagnostic delay (n = 687) | **0.194** | (-0.264, 0.054) | 0.449 | (-0.228, 0.101) |  |  |
| Health-related behavior | | | | | | |
| Smoking (n = 696) | 0.614 | (-9.327, 5.517) |  |  |  |  |
| PE (n = 693) | **0.197** | (-2.526, 12.224) | **0.011** | (2.385, 17.997) | **0.01** | (2.457, 18.056) |
| Living conditions |  |  |  |  |  |  |
| Living area >5years (rural/urban) (n = 635) | 0.403 | (-11.965, 4.815) |  |  |  |  |
| Living area in the last 5 years (rural/urban) (n = 646) | **0.187** | (-13.435, 2.629) | 0.289 | (-13.017, 3.879) |  |  |
