# Supplementary material for: Analysis of routine blood parameters in patients with amyotrophic lateral sclerosis and evaluation of a possible correlation with disease progression—a multicenter study
Source: Front Neurol. 2022 Jul 27;13:940375. doi: 10.3389/fneur.2022.940375 (PMC9364810; doi:10.3389/fneur.2022.940375)
Supplement: Supplementary file 5 [file Table_5.DOCX]

Supplemental Table 5 Correlation of disease characteristics/living conditions and HDL cholesterol level

|  | Univariat analysis | | Multivariate analysis (n = 565) | | Multivariate analysis backward selection | |
| --- | --- | --- | --- | --- | --- | --- |
|  | *p* value | 95% CI | *p* value | 95% CI | *p* value | 95% CI |
| Basics | | | | | | |
| Gender  (n = 620) | **<0.001** | (-12.747, -7.133) | **<0.001** | (-13.613, -7.417) | **<0.001** | (-14.412, -8.438) |
| Age at diagnosis (n = 601) | **0.003** | (0.059, 0.285) | **0.044** | (0.003, 0.232) | **0.024** | (0.017, 0.24) |
| Statin intake (n = 618) | 0.886 | (-3.645, 4.222) |  |  |  |  |
| Disease characteristics | | | | | | |
| Limb onset  (n = 620) | Reference |  | Reference |  |  |  |
| Bulbar onset (n = 620) | **0.064** | (-0.178, 6.484) | 0.728 | (-3.23, 4.618) |  |  |
| Thoracic onset (n = 620) | **0.155** | (-2.675, 16.724) | 0.152 | (2.588, 16.561) |  |  |
| Dyscognition (n = 620) | 0.654 | (-12.405, 19.737) | 0.777 | (-17.595, 23.53) |  |  |
| Predominant UMN (n = 590) | 0.894 | (-3.08, 3.527) |  |  |  |  |
| Predominant LMN (n = 590) | **0.122** | (-6.808, 0.806) | 0.229 | (-6.295, 1.508) |  |  |
| Upper limb (n = 620) | **0.108** | (-5.414, 0.539) | 0.902 | (-3.167, 3.589) |  |  |
| Lower limb (n = 620) | 0.237 | (-4.754, 1.178) |  |  |  |  |
| Diagnostic delay (n = 601) | **0.097** | (-0.112, 0.009) | 0.066 | (-0.119, 0.004) |  |  |
| Health-related behavior | | | | | | |
| Smoking (n = 614) | **0.031** | (-6.11, -0.296) | 0.078 | (-5.871, 0.317) |  |  |
| PE (n = 612) | **0.008** | (1.004, 6.83) | **<0.001** | (3.534. 9.619) | **<0.001** | (2.772, 8.709) |
| Living conditions |  |  |  |  |  |  |
| Living area >5years (rural/urban) (n = 557) | 0.774 | (-3.819, 2.845) |  |  |  |  |
| Living area in the last 5 years (rural/urban) (n = 582) | 0.951 | (-3.087, 3.285) |  |  |  |  |
