# Supplementary material for: Analysis of routine blood parameters in patients with amyotrophic lateral sclerosis and evaluation of a possible correlation with disease progression—a multicenter study
Source: Front Neurol. 2022 Jul 27;13:940375. doi: 10.3389/fneur.2022.940375 (PMC9364810; doi:10.3389/fneur.2022.940375)
Supplement: Supplementary file 6 [file Table_6.DOCX]

Supplemental Table 6 Correlation of disease characteristics/living conditions and LDL cholesterol level

|  | Univariat analysis | | Multivariate analysis (n = 553) | | Multivariate analysis backward selection | |
| --- | --- | --- | --- | --- | --- | --- |
|  | *p* value | 95% CI | *p* value | 95% CI | *p* value | 95% CI |
| Basics | | | | | | |
| Gender  (n = 615) | **<0.001** | (-19.073, -5.619) | **0.003** | (-19.026, -4.038) | **<0.001** | (-20.586, -6.141) |
| Age at diagnosis (n = 598) | 0.771 | (-0.214, 0.314) |  |  |  |  |
| Statin intake (n = 613) | **0.068** | (-17.659, 0.623) | 0.234 | (-15.196, 3.719) |  |  |
| Disease characteristics | | | | | | |
| Limb onset  (n = 615) | Reference |  | Reference |  |  |  |
| Bulbar onset (n = 615) | 0.907 | (-7.298, 8.217) | 0.345 | (-13.6, 4.759) |  |  |
| Thoracic onset (n = 615) | **0.056** | (-0.552, 44.566) | 0.109 | (-4.344, 42.986) |  |  |
| Dyscognition (n = 615) | 0.215 | (-60.995, 13.754) | 0.209 | (-62.141, 13.6) |  |  |
| Predominant UMN (n = 586) | 0.609 | (-9.708, 5.691) |  |  |  |  |
| Predominant LMN (n = 586) | 0.374 | (-4.878, 12.945) |  |  |  |  |
| Upper limb (n = 615) | **0.092** | (-12.871, 0.966) | 0.086 | (-15.2, 1.009) |  |  |
| Lower limb (n = 615) | 0.899 | (-6.497, 7.396) |  |  |  |  |
| Diagnostic delay (n = 598) | **0.113** | (-0.253, 0.027) | 0.127 | (-0.261, 0.032) |  |  |
| Health-related behavior | | | | | | |
| Smoking (n = 609) | **0.068** | (-13.082, 0.475) | 0.216 | (-12.189, 2.759) |  |  |
| PE (n = 607) | **0.095** | (-1.007, 12.602) | **0.005** | (3.12, 17.65) | **0.01** | (2.316, 16.618) |
| Living conditions |  |  |  |  |  |  |
| Living area >5years (rural/urban) (n = 557) | 0.234 | (-12.523, 3.065) |  |  |  |  |
| Living area in the last 5 years (rural/urban) (n = 580) | **0.107** | (-13.534, 1.319) | 0.259 | (-11.996, 3.238) |  |  |
