# Supplementary material for: Analysis of routine blood parameters in patients with amyotrophic lateral sclerosis and evaluation of a possible correlation with disease progression—a multicenter study
Source: Front Neurol. 2022 Jul 27;13:940375. doi: 10.3389/fneur.2022.940375 (PMC9364810; doi:10.3389/fneur.2022.940375)
Supplement: Supplementary file 7 [file Table_7.DOCX]

Supplemental Table 7 Correlation of disease characteristics/living conditions and triglyceride level

|  | Univariat analysis | | Multivariate analysis (n = 630) | | Multivariate analysis backward selection | |
| --- | --- | --- | --- | --- | --- | --- |
|  | *p* value | 95% CI | *p* value | 95% CI | *p* value | 95% CI |
| Basics | | | | | | |
| Gender  (n = 668) | **<0.001** | (11.477, 32.258) | **<0.001** | (9.451, 31.985) | **<0.001** | (12.305, 33.695) |
| Age at diagnosis (n = 649) | 0.618 | (-0.519, 0.309) |  |  |  |  |
| Statin intake (n = 665) | **0.037** | (0.872, 29.093) | **0.009** | (4.998, 34.109) | **0.008** | (4.99, 33.585) |
| Disease characteristics | | | | | | |
| Limb onset  (n = 669) | Reference |  | Reference |  |  |  |
| Bulbar onset (n = 668) | **0.055** | (-23.473, 0.269) | 0.438 | (-19.946, 8.647) |  |  |
| Thoracic onset (n = 668) | 0.824 | (-31.211, 39.211) | 0.738 | (-29.345, 41.375) |  |  |
| Dyscognition (n = 668) | 0.45 | (-76.362, 33.879) | 0.742 | (-80.811, 57.602) |  |  |
| Predominant UMN (n = 637) | 0.344 | (-17.557, 6.129) |  |  |  |  |
| Predominant LMN (n = 637) | **0.019** | (2.677, 29.401) | **0.058** | (-0.457, 27.717) | **0.024** | (2.056, 28.751) |
| Upper limb (n = 668) | 0.42 | (-15.196, 6.345) |  |  |  |  |
| Lower limb (n = 668) | **0.059** | (-0.409, 21.032) | 0.141 | (-3.064, 21.425) | **0.043** | (0.341, 22.052) |
| Diagnostic delay (n = 649) | 0.51 | (-0.149, 0.3) |  |  |  |  |
| Health-related behavior | | | | | | |
| Smoking (n = 661) | **0.013** | (2.839, 23.883) | 0.31 | (-5.271, 16.873) |  |  |
| PE (n = 658) | 0.541 | (-13.872, 7.279) |  |  |  |  |
| Living conditions |  |  |  |  |  |  |
| Living area >5years (rural/urban) (n = 605) | 0.714 | (-14.289, 9.789) |  |  |  |  |
| Living area in the last 5 years (rural/urban) (n = 628) | 0.254 | (-18.281, 4.839) |  |  |  |  |
