# Supplementary material for: Analysis of routine blood parameters in patients with amyotrophic lateral sclerosis and evaluation of a possible correlation with disease progression—a multicenter study
Source: Front Neurol. 2022 Jul 27;13:940375. doi: 10.3389/fneur.2022.940375 (PMC9364810; doi:10.3389/fneur.2022.940375)
Supplement: Supplementary file 8 [file Table_8.DOCX]

Supplemental Table 8.A Linear regression models for **rate of decay** and blood parameters (Germany).

|  | Univariat analysis | | | Multivariate analysis | | | Rate of decay ↑ |
| --- | --- | --- | --- | --- | --- | --- | --- |
| Variable | n | *p* value | 95% CI | n | *p* value | 95% CI |  |
| CK | 344 | **0.127** | (-0.001, 0.000) | 344 | 0.412 | (-0.001, 0.000) |  |
| Albumin | 168 | 0.212 | (-0.082, 0,365) | 168 | 0.372 | (-0.125, 0.333) |  |
| Creatinine | 353 | 0.21 | (-0.321, 0.071) | 353 | 0.356 | (-0.285, 0.103) |  |
| Total cholesterol | 214 | 0.675 | (-0.001, 0.002) | 214 | 0.688 | (-0.001, 0.002) |  |
| HDL | 172 | 0.889 | (-0.005, 0.004) | 172 | 0.395 | (-0.006, 0.003) |  |
| LDL | 178 | 0.618 | (-0.002, 0.003) | 178 | 0.53 | (-0.002, 0.003) |  |
| Triglyceride | 196 | **0.013** | (0.000, 0.003) | 176 | 0.005 | (0.001, 0.003) | TG ↑ |

Controlled for gender, age at diagnosis, diagnostic delay, region of onset, UMN or LMN predominance.
