# Supplementary material for: Analysis of routine blood parameters in patients with amyotrophic lateral sclerosis and evaluation of a possible correlation with disease progression—a multicenter study
Source: Front Neurol. 2022 Jul 27;13:940375. doi: 10.3389/fneur.2022.940375 (PMC9364810; doi:10.3389/fneur.2022.940375)
Supplement: Supplementary file 9 [file Table_9.DOCX]

Supplemental Table 8.B Linear regression models for **rate of decay** and blood parameters (Portugal).

|  | Univariat analysis | | | Multivariate analysis | | | | Rate of decay ↑ |
| --- | --- | --- | --- | --- | --- | --- | --- | --- |
| Variable | n | *p* value | 95% CI | n | *p* value | 95% CI |  | |
| CK | 201 | 0.055 | (-0.001, 0.000) | 201 | 0.745 | (-0.001, 0.001) |  | |
| Albumin | 157 | 0.983 | (-0.487, 0.498) | 157 | 0.502 | (-0.317, 0.644) |  | |
| Creatinine | 280 | 0.054 | (-0.012, 1.356) | 280 | 0.224 | (-0.27, 1.149) |  | |
| Total cholesterol | 218 | 0.057 | (-0.006, 0.000) | 218 | 0.126 | (-0.005, 0.001) |  | |
| HDL | 172 | 0.103 | (-0.015, 0.0ß01) | 172 | 0.061 | (-0.016, 0.000) |  | |
| LDL | 171 | 0.059 | (-0.007, 0.000) | 171 | 0.108 | (-0.006, 0.001) |  | |
| Triglyceride | 201 | 0.085 | (0.000, 0.004) | 201 | **0.036** | (0.000, 0.004) | TG ↑ | |

Controlled for gender, age at diagnosis, diagnostic delay, region of onset, UMN or LMN predominance.
